# Goal-directed fluid therapy based on noninvasive cardiac output monitor reduces postoperative complications in elderly patients after gastrointestinal surgery: A randomized controlled trial

**DOI:** 10.12669/pjms.346.15854

**Published:** 2018

**Authors:** Kaiyu Yin, Jiahui Ding, You Wu, Mingqing Peng

**Affiliations:** 1 *Kaiyu Yin, Department of Anesthesiology, Yongchuan Hospital of Chongqing Medical University, Chongqing, 402160, China*; 2 *Jiahui Ding, Department of Anesthesiology, Yongchuan Hospital of Chongqing Medical University, Chongqing, 402160, China*; 3 *You Wu, Department of Anesthesiology, Yongchuan Hospital of Chongqing Medical University, Chongqing, 402160, China*; 4 *Mingqing Peng, Department of Anesthesiology, Yongchuan Hospital of Chongqing Medical University, Chongqing, 402160, China*

**Keywords:** Goal-directed fluid therapy, Noninvasive Cardiac Output Monitor, Gastrointestinal surgery, Elderly, Postoperative complication

## Abstract

**Objective:**

Goal-directed fluid therapy (GDFT) was associated with improved outcomes after surgery. Noninvasive Cardiac Output Monitoring (NICOM) has proved to be a good choice for guiding GDFT. This study evaluated the effect of GDFT based on NICOM on prognosis in elderly patients undergoing resection of gastrointestinal tumor.

**Methods:**

Fifty patients scheduled for elective laparoscopic radical resection for stomach, colon or rectal cancer in Yongchuan Hospital of Chongqing Medical University between November 2014 and December 2015 were included and randomly divided into two groups: conventional fluid therapy (group C, n=25) and goal-directed fluid therapy (group G, n=25). The primary outcome was moderate or severe postoperative complications within 30 days.

**Results:**

Finally, 45 patients successfully completed the study (group G, n=22; group C, n=23). There were no difference of the duration of surgery, the requirement of vasoactive agents and the bleeding volume between two groups (*P*>0.05). Total fluids infused were 2956±629 ml (group C) and 2259±454 ml (group G) (*P*<0.05), while the requirement of colloid was increased in group G (1103±285ml vs 855±226ml) (*P*<0.05). The MAP and the mean CI were higher in group G (*P*<0.05). Compared with group C, the time when the patients passed the flatus and the length of hospital stay after operation were shortened in group G (12.6±2.4day vs17.2±2.6day), the incidence of postoperative complications were significantly lower in group G (*P*<0.05).

**Conclusions:**

Goal-directed fluid therapy based on NICOM was significantly associated with improvement of prognosis in elderly patients undergoing resection of gastrointestinal tumor which reduced postoperative complications.

## INTRODUCTION

Intraoperative fluid management is difficult in anesthesia for elderly patients with gastrointestinal tumor. The conventional liquid treatment scheme is based on patient’s weight or determined by the experience of anesthesiologist, unable to meet individual needs. Elderly patients with poor compensation often suffer from volume overload or insufficient blood circulation, resulting in systemic edema or organ dysfunction, increasing incidence of postoperative complications.^1^,^2^ Concept of Goal-directed fluid therapy (GDFT) is proposed to solve this problem. GDFT is a fluid treatment strategy for personalized infusion according to the general condition of patients and the volume status, the strategy can optimize the hemodynamic of the patients and let the cardiac output and oxygen supply reach extraordinary values, improving the prognosis of patients.^3^,^4^ However, most hemodynamic monitors used widely now are invasive, which may cause complications such as hematoma, permanent occlusion, bleeding, sepsis^5^, and bring discomfort to the patients. NICOM is an application of biological reactor technology, monitoring cardiac output (CO), cardiac index (CI), stroke volume (SV), stroke volume variability (SVV) and other hemodynamic parameters by analyzing electric pulses through the frequency change of chest. It is sensitive, accurate and in good agreement with pulmonary artery catheter (PAC), Trans-Esophageal Doppler (TED), Pulse-induced Continuous Cardiac Output (PICCO) and Vigileo monitoring technologies, a number of studies have confirmed.^6^-^8^ Compared with these invasive or minimally invasive techniques, the NICOM technology is safer for patients and easier to operate for the anesthesiologists.

We carried out this study to investigate the effect of different fluid therapies on gastrointestinal surgery in elderly patients. We hypothesized that it might stable hemodynamics, promote the recovery of gastrointestinal function and reduce postoperative complications as in previous studies.

## METHODS

This research study was approved by The Ethics Committee of Yongchuan Hospital of Chongqing Medical University, and was performed only after informed consent was signed by the detailed known patients and their families. At last, 50 qualified patients (aged 65-90 years, with a body mass index (BMI) between 18-28 kg.m^-2^ and an American Society of Anesthesiology classification of II-III, normal liver and renal function) with laparoscopic gastrointestinal surgery in Yongchuan Hospital of Chongqing Medical University between November 2014 and December 2015 were divided into two groups by random number table method. Numbers were concealed in sealed envelopes and it would not be opened before the induction of anesthesia.

### Study Procedure

Two groups of patients were not treated with premedication but regular fasting. While in operation room, routine monitoring of heart rate (HR), blood pressure (BP), electrocardiogram (ECG), oxygen saturation (SpO_2_), opened venous access, finished catheterization of radial artery and internal jugular vein after local anesthesia and monitored MAP, CVP.

All the patients were treated by the same anesthesiologists and the same anesthesia scheme. Anesthesia was induced by intravenous injection of midazolam 0.05 mg/Kg, sufentanil 0.3-0.4 ug/Kg, etomidate 0.3 mg/Kg and vecuronium 0.1mg/Kg. We used mechanical ventilation after oxygen mask inhalation and tracheal intubation, the anesthesia machine parameters were maintained: tidal volume (VT) 8-10 ml/Kg; respiratory rate (RR) 10-12/min, inspiratory/expiratory ratio 1:2; PET_CO2_ (the end-tidal CO_2_ pressure) 35~45mmHg. As for the maintaining of anesthesia: 1%~3% Sevoflurane inhalation; continuous infusion of remifentanil 0.1~0.2 ug.Kg^-1^.min^-1^, propofol 50~100 ug.Kg^-1^.min^-1^. During the operation, the blanket and continuous heating device were used to maintain the patient’s body temperature to ensure not lower than 36 degrees Celsius, and the bispectral index (BIS) was monitored to maintain the value between 40-60.

Group C was treated by classical fluid infusion scheme, the total volume included compensatory volume expansion, cumulative loss, physiological demand, continuous loses and the third clearance loss. Infusion rate and dose in operation were adjusted in a timely manner according to the MAP, CVP and urine volume. The specific program: If MAP 65-90 mmHg, CVP 6~12cmH_2_O, urine volume was more than 0.5 ml.Kg^-1^.h^-1^, there was no need to deal with; If CVP> 12 cmH_2_O and MAP>90 mmHg, controlled the speed of infusion and used vasodilators or appropriately deepened anesthesia according to the BIS value; If MAP<65 mmHg and it was suitable for using dobutamine and accelerating the infusion speed; If CVP<6 cmH_2_O and MAP<65 mmHg, then added 6% hydroxyethyl starch 130/0.4 250ml, if the goal was not achieved, continued to infuse 6% hydroxyethyl starch 130/0.4 250ml; If CVP<6 cmH_2_O but MAP more than 65 mmHg, appropriately adjusted the depth of anesthesia according to the BIS value as well as supplied with 6% hydroxyethyl starch 130/0.4 250ml.

Group G adopted the GDFT programme: When the CI was in 2.5-4.0 L.min^-1^.m^-2^ and SVV<13%, no treatment; When CI>4.0 L.min^-1^.m^-2^, controlled the speed of infusion and used vasodilators or appropriately adjusted the depth of anesthesia according to the BIS value; When the CI <2.5 L.min^-1^.m^-2^ and SVV<13%, took advantage of dobutamine; When CI<2.5 L.min^-1^.m^-2^ and SVV>13%, 6% hydroxyethyl starch injection 130/0.4 250ml was infused in 5~15 minutes.

The two groups used dobutamine (drug concentration 50mg/50ml) at the speed of 2.5 ug.kg^-1^.min^-1^, and the background infusion volume was 8ml.kg^-1^.h^-1^ of compound sodium chloride and input red blood cell suspension when Hb<80 g/L if they needed. Postoperative analgesia of patients were both (sufentanil 1 g/kg+ dezocine 0.3 mg/kg+ ondansetron 8mg).

Patients were continuously monitored regarding conventional hemodynamic parameters every 10 minutes. The NICOM system was used to obtain CI, SVV and other hemodynamic parameters. Hemodynamic indexes were also recorded at the following time points: the onset of the monitoring (T_1_) and the end of the surgery (T_2_); Also, the amount of intraoperative input and output volume as well as the use of vasoactive drugs were recorded. We recorded patients’ undergoing collides volumes, crystal volumes, blood losses and perioperative urine outputs as well.

All the patients were treated by the same surgeon group, and the intraoperative fluid management program was blind to them. The primary outcome was moderate or severe postoperative complications within 30 days. Secondary endpoints were return of gastrointestinal function and the length of hospital stay after operation. The extubation time, intraoperative hemodynamic parameters and the use of vasoactive agents were also recorded.

### Statistical analysis

According to previous and relevant statistical data, a prior power calculation showed that 25 patients would be required in each group to detect 20 percents difference in the SRS (simple random sample) with type I error of 0.05 and type II error of 0.2. Thus a sample size of 50 was assessed.

Statistical analyses were performed using SPSS version 18.0 statistical software with intention to treat, measurement data was presented by mean ± standard deviation (x ±S), comparison in the group used analysis of variance of repeated measurement, comparison between groups used *t* test, count data were compared using the *χ*^2^ test. When *P* < 0.05, the difference was considered to be statistically significant.

## RESULTS

A total of fifty patients undergoing elective colorectal resection were randomized (25 per group), five failed to complete the study. There were no significantly statistical differences in sex ratio, age, body mass index (BMI), ASA classification, hemoglobin, preoperative complications, operation time and pneumoperitoneum time between the two groups (*P*>0.05) (Table-I).

**Table-I:** Patients characteristics and clinical data of two groups (*x̄*±S).

Variable	Control group (group C, n=23)	GDFT group (group G, n=22)
number of cases	23	22
Male / female	10/13	11/11
Age (year)	68.3±5.8	69.4±6.4
BMI(Kg.m^-2^)	22.6±2.2	22.4±2.4
ASA(II/III)	12/13	11/14
COPD	10	12
Hypertension	19	18
Coronary heart disease	9	8
Diabetes mellitus	8	7
Cerebrovascular disease	3	5
Other complications	5	3
Preoperative Hb (g/L)	122.0±13.3	120.0±12.4
operation time (h)	3.3±0.7	3.4±0.6
Time of pneumoperitoneum (h)	3.0±0.8	3.1±0.9

*Note:* compared with group C, P>0.05.

### Comparison of intraoperative liquid intake and vasoactive drugs in two groups

Compared with group C, the total amount of intravenous infusion and the amount of crystal infusion decreased significantly in group G, but the amount of colloid increased obviously. There was no significant difference in urine volume, blood loss and transfusion volume (Table-II).

**Table-II:** Fluid intake and use of vasoactive drugs in the two groups (*x̄*±S).

Group	Case	Total infusion (ml)	Colloidal amount (ml)	Crystal amount (ml)	Blood loss (ml)	Blood transfusion volume (ml)	Urine volume (ml)	Use of vasoactive agents (case)
Group C	23	2956±629	855±226	1771±450	315±78	156±67	386±96	16
Group G	22	2259±454*	1103±285*	1006±250*	298±67	160±54	378±92	18

*Note:* compared with group C,

* P < 0.05, significantly different between groups.

### Comparison of hemodynamic parameters between two groups

Compared with the group C, T_1_ of the two groups about MAP, HR, CVP and CI in group G had no significant difference; Compared with T_1_, two groups of CVP and CI were significantly increased in T_2_, however, blood lactate content in group C increased significantly. Compared with group C, ScvO_2_ in group G was significantly increased and the content of blood lactic acid decreased significantly when it came to T2 (*P*<0.05) (Table-III).

**Table-III:** Comparison of relative indexes of T_1_ and T_2_ time points in two groups (*x̄*±S).

	T_1_		T_2_	

Index	Group C (23)	Group G (22)	Group C (23)	Group G (22)
MAP(mmHg)	73.2±8.3	72±7.9	75.4±9.1	78.5±9.6
HR(bpm)	79.3±7.6	81±6.8	76.5±8.4	75.5±7.4
CI(L.min-^1^•m-^2^)	2.5±0.6	2.4±0.7	2.7±0.4*	2.8±0.5*
CVP(cmH_2_O)	5.8±1.6	5.9±1.7	7.8±1.6*	8.5±1.4*
SVV(%)	-	-	10.1±1.7	8.9±1.4
ScvO2(%)	71.4±5.4	70.9±6.2	70.5±5.6	73.8±3.7^#^
Lac(mmol/L)	1.1±0.3	1.2±0.4	1.3±0.5	1.0±0.2^#^

*Note:* compared with T_1_,

* P < 0.05; compared with group C,

# P < 0.05

### Comparison of postoperative conditions between the two groups

Compared with group C, group G had earlier exhaust time, shorter hospitalization time, less surgical related complications, pulmonary and cardiovascular complications; Two groups had no statistically significant differences in extubation time, urinary system complications. (Table-IV).

**Table-IV:** Functional recovery and incidence of postoperative complications.

Group	Case	Tracheal extubation time (h,±S)	The first exhaust time (h,±S)	Surgical related complication (%)	Pulmonary (%)	Cardiovascular (%)	Urinary system (%)	Postoperative hospital stay(d,±S)
Group C	23	0.68±0.25	86±23	38	40	32	8	17.2±2.6
Group G	22	0.61±0.23	72±24*	19*	18*	14*	10	12.6±2.4*

*Note:* compared with group C,

* P < 0.05

## DISCUSSION

The main finding of our study was that patients in group G received more intraoperative colloid and smaller volume of intravenous fluid overall. Compared with group C, CVP, ScvO_2_ and MAP were higher at T_2_, but blood lactate content was lower at T_2_. Thus these patients were likely to have more stable dynamics and good tissue perfusion. This might translate into some differences in surgical recovery and clinical outcome that patients in group G had fewer postoperative complications.

Fluid management is an important part of clinical anesthesia, and has also been a controversial hot topic among scholars. For anesthesiologists, the key to fluid therapy is the timely and accurate diagnosis of patient blood volume status so that they can take measures instantly and effectively to prevent organ damage, thereby, improving the prognosis of patients.^9^ Nevertheless, conventional hemodynamic indexes such as BP (blood pressure), HR and CVP are static and lagging^10^,^11^, affecting the judgment of patients’ effective circulating blood volume, especially in elderly patients. In this study, most fluid challenge time of group G patients was in the first half of the total operation time. But patients in group C might be a low blood volume and not awarded for a long time due to lag of conventional monitor indicators, leading to tissue ischemia and hypoxia, affecting postoperative healing and gastrointestinal function recovery. On the contrary, early colloidal challenge treatment in the group G seemed to improve hemodynamic stability so that the first exhaust time was earlier, the hospital stay after operation was shorter.

As a bright star of fluid management, with the use of fluid load or combination of inotropic drugs to make body achieve the best oxygenation, GDFT can improve capacity state timely and dynamically. The strategy was proved to optimize cardiac preload, meet individual needs, and was important in maintaining effective blood volume and reducing the incidence of postoperative complications.^3^-^4^,^12^-^14^ Studies have showed that SVV had a good correlation with capacity change,^15^,^16^ we can objectively and instantly assess where the position of patients cardiac function is on the Frank-Starling curve, so as to understand its capacity and realize the implementation of individualized rehydration. SVV was recommended as a standard treatment strategy for major surgery. At the same time, CI could effectively assess the oxygen supply of the organism under the normal condition of arterial oxygen saturation and hemoglobin^17^, so this study took SVV<13% and CI between 2.5-4.0 as the target to carry out GDFT.

The monitoring methods of GDFT are diverse, but NICOM is more and more popular for its completely non-invasive, awake patients tolerated and simple operation. It can monitor kinetic parameters such as SVV, CI and SV continuously and dynamically, the monitoring accuracy was confirmed by many studies.^6^-^8^ Waldron^6^ and other studies showed that there was good correlation between the data of NICOM and esophageal doppler monitor (EDM), and the missing data was less than EDM.

Patients characteristics and clinical data of two groups showed no significant difference, indicating the factors of two groups patients before surgery were consistent; Anesthesia methods, carbon dioxide pneumoperitoneum time, operation time and bleeding volume were not significantly different in two groups of patients, which roughly implied that the impact of anesthesia and operation on patients physiology were equal. Therefore, the two groups were comparable. The study results suggested that, MAP and the average CI in the group G was higher than that of group C, contrarily, the average SVV was lower than the group C. We could speculate that central function of group G patients was closer to the Frank-Starling curve of platform level the effect of fluid treatment on the group G was better than group C to maintain hemodynamic stability. Meanwhile, ScvO_2_ at T_2_ of group G increased obviously higher than that of group C, and the content of blood lactic acid in group G decreased evidently, which might confirm the above inference. When the body meets with insufficient perfusion and hypoxia, it often leads to metabolic disorder and the increase of blood lactate content^18^. ScvO_2_ could quickly reflect the body’s oxygenation, detect tissue hypoxia early, and ScvO_2_ was proved to have good correlation of mixed venous oxygen saturation (SVO_2_) measured by pulmonary artery catheter,^18^-^19^ but ScvO_2_ measurement was safer, faster and more convenient, so the use of ScvO_2_ was more popular in clinical. Thus it can be seen that the difference of ScvO_2_ and blood lactic acid content between the two groups showed that the GDFT guided by NICOM was more conducive to maintain the balance of oxygen supply and demand to improve microcirculation.

### Limitations of the study

It was a relatively small trial and therefore easy to confounding from factors that we could not control. We made no attempt to regulate postoperative i.v.fluid therapy, and this might have influence on the effect of intraoperative therapy. Further studies are necessary, likewise, whether GDFT based on NICOM can be used in conscious patients to get clinical benefits during operation. A definitive, large effective trail is required.

In conclusion, we found that GDFT based on NICOM was associated with shorter hospital stay, faster recovery of gastrointestinal function, less postoperative complications and better hemodynamic indices than traditional fluid therapy. It may improve prognosis for the elderly patients undergoing gastrointestinal surgery. However, it was not associated with decrease in complications of urinary system.

### Authors’ Contributions

**MP:** Designed the study, analyzed data and revised the manuscript;

**KY:** Performed the patients’ recruitment, randomization and prepared the manuscript;

**JD:** Performed anesthesia care, revised the manuscript, participated on data analysis

**YW:** Responsible for collecting data and postoperative clinical assessment and revised the manuscript;

All authors have approved the final version to be published.
